# Brain Mapping and Synapse Quantification *In vivo*: It's Time to Imaging

**DOI:** 10.3389/fnana.2017.00017

**Published:** 2017-03-07

**Authors:** Angel Acebes

**Affiliations:** Department of Basic Medical Sciences, Centre for Biomedical Research of the Canary Islands, Institute of Biomedical Technologies, University of La LagunaLa Laguna, Spain

**Keywords:** brain mapping, MRI, cortex neuroanatomy, [^11^C]UCB-J radioligand, *in vivo* synapse quantification

## Background

Two papers have rattled our summer this year, bringing us exciting news to the field. In the first one, Glasser and colleagues, using a neuroanatomical approach and employing multimodal magnetic resonance images (MRI), were able to define 180 areas per brain hemisphere considering cortical architecture, functionality, connectivity, and topography in healthy young adults (Glasser et al., 2016a). This study provides higher precision about human cerebral cortex neuroanatomy and, more interestingly, paves the way to future assessments of individual variations related to development, aging, and diseases. In the second one, Finnema and collaborators reported the use of a synaptic vesicle glycoprotein radioligand combined with positron emission tomography (PET) to quantify synaptic density in living human brains (Finnema et al., 2016). Remarkably, the possibility to perform *in vivo* synaptic quantifications represents a powerful tool in the diagnosis of synaptic changes and synapse loss which are early features directly associated with numerous brain disorders. In this Opinion paper, I will contribute an evaluation of these articles, comparing them with alternative approaches in the field and aiming to foresee future research directions.

## Unambiguously mapping the human brain, not wishful thinking?

In 1536, the Spanish sailor Andrés de Urdaneta accomplished a world circumnavigation sailing practically blinded. He did not have creditable maps and relied on a few rudimentary drawings that barely indicated territories, capes and gulfs from the shores, performed by former XV–XVI centuries Spanish and Portuguese navigators. Over one century later, the situation was analogous to the first neuroanatomists facing the challenge to characterize neuronal connectivity in anatomically segregated brain regions, helped only by the first Cajal neuronal drawings (DeFelipe, 2006; De Carlos and Borrell, 2007, Figure 1A). From a neuroanatomical perspective, they started navigating through the brain territories and shores as blinded as Urdaneta did.

**Figure 1:**
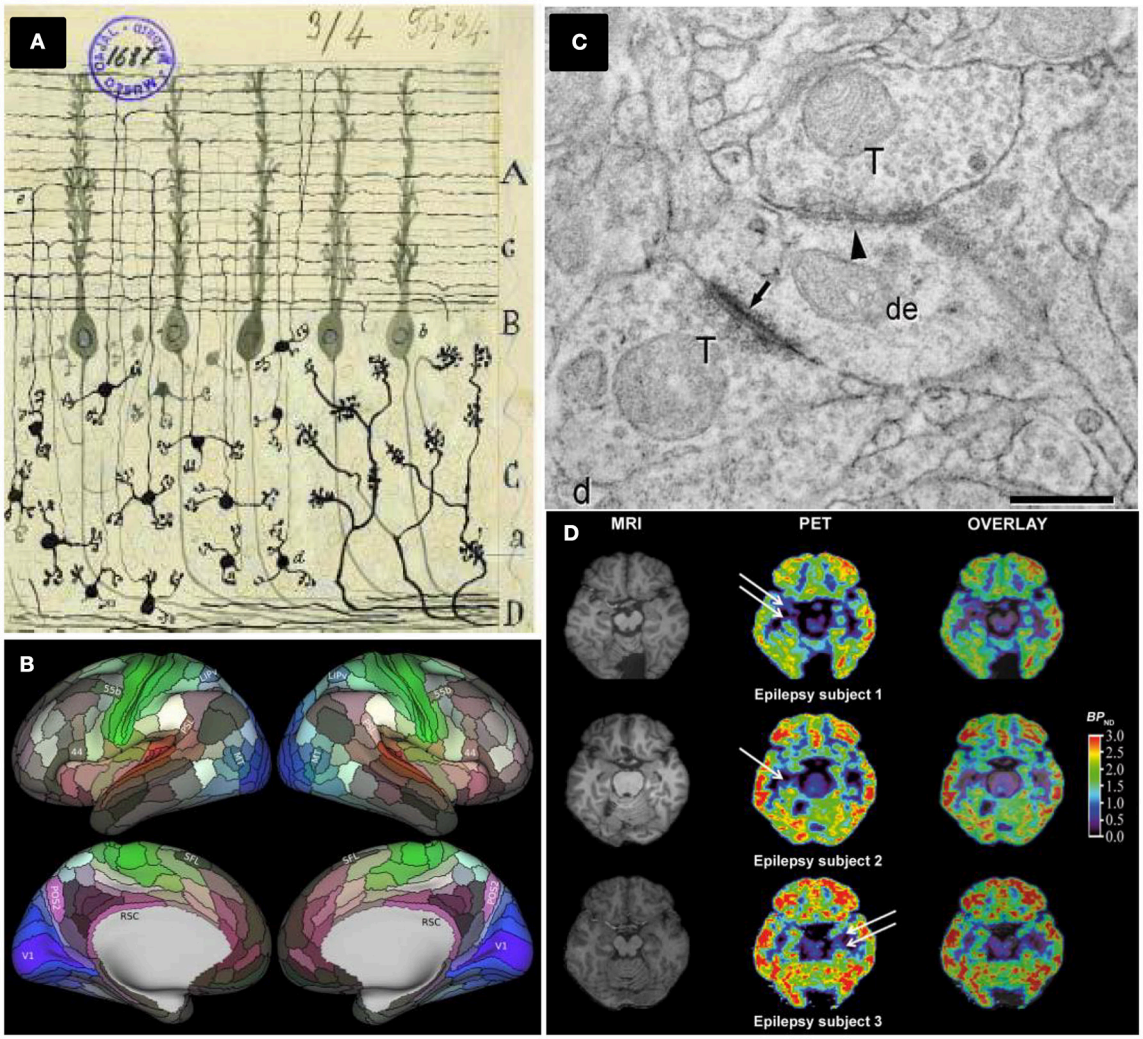
**Different brain mapping and synapse counting methods. (A)** Neurons and brain maps. A reproduction of a Cajal original drawing showing mossy fibers entering (a) and arborizing in the granular layer whereas granule cells (d) send ascending axons that bifurcate (e) in the molecular layer. Adapted by permission from Macmillan Publishers Ltd: De Carlos and Borrell (2007). **(B)** The HCP's multimodal parcellation, version 1.0: 180 areas delineated and identified in both left and right hemispheres are displayed on inflated cortical surfaces. Glasser et al. (2016a), with permission from Elsevier. **(C)** Synapses ultrastructure: Asymmetric synapses (arrows) show a prominent postsynaptic density, whereas symmetric synapses (arrowhead) present a thin postsynaptic density: de, dendritic shaft; ds, dendritic spines; T, axon terminals, scale bar 0.4 μm. Permission granted for the use of the figure corresponding to Alonso-Nanclares et al. (2008) Nacional Academy of Sciences, U.S.A. **(D)** PET evaluation with [^11^C]UCB-J reveals synapse loss in epilepsy patients: white arrows indicate loss of [^11^C]UCB-J binding in the mesial temporal lobe. From Finnema et al. (2016). Reprinted with permission from AAAS.

The identification and deep characterization of anatomical brain subdivisions represent a crucial task to understand how the brain works. An accurate parcellation would provide a map to elucidate which brain areas interact together. This scientific and technical challenge has leaped an extraordinary step forward with the approach chosen by Glasser and collaborators, applying new- and also improved- methods to analyse high-quality magnetic resonance imaging (MRI) data from the Human Connectome Project (HCP). The HCP started in 2010 when two NIH consortia developed a series of neuroimaging methods acquiring neuroimaging, behavioral and genotype data sets of unprecedented size and quality. These data were obtained from around 1,100 healthy young adults, aiming to map the normal human brain connectome (Glasser et al., 2016b).

Taking advantage of major advances in image acquisition and pre-processing, Glasser and collaborators carried out a global analysis gathering architecture, function, connectivity, and topographical properties across the neocortex of both brain hemispheres, in a precisely aligned set of 210 healthy young adults. Their results are striking, yielding a major advance of reproducible brain images with respect to former human cortical parcellations, by delineating a total of 180 cortical areas (97 new areas and 83 areas previously reported) per brain hemisphere (Glasser et al., 2016a, Figure 1B). The identification of these cortical areas opens research avenues to study human cognition, development and aging. In addition, it provides new approaches to face diseases helping to characterize possible changes linked to learning or cognitive disabilities. Furthermore, the access to detailed and individualized maps of cortical areas in living humans by non-invasive MRI methods have profound implications in how brain surgery will be performed in the future.

I will highlight some facts to consider this paper as strongly relevant:

1. One major problem of unambiguously identifying brain areas as anatomically and functionally distinct has been the very definition of an area. From my point of view, this study provides solid and accurate grounds to solve that issue.
2. Most previous studies concerning brain parcellations have relied on stained micro-sectioning of the brain. In contrast, Glasser's method relies purely on magnetic resonance images. It is therefore easily replicable, precise and accurate. In addition, this new method is fully automated to detect 96.6% of individual cortical areas in new subjects.
3. Previous studies of brain parcellations have been based on a single property of neurological tissue (e.g., cell distribution). By contrast, this study considers brain architecture, functionality, connectivity, and topographical properties.
4. This study combines two types of multimodal magnetic resonance images, T1 and T2. In preceding studies, only T1 images are used whereas the T2 images provide information about myelin content.
5. This work identifies new cortical areas that were undetected by other approaches (e.g., Brodmann's hand drawn parcellation published in 1909), providing a wealth of new information about brain topography.
6. Furthermore, this study points out atypical topological arrangements of some areas in some individuals, discernible across multiple modalities, including resting-state networks, task-fMRI activations, and myelin maps. This individual variability boosts intriguing questions for future exploration.
7. These new data and tools will be freely accessible to neuroanatomists on GitHub (https://github.com/), the HCP (http://humanconnectome.org) and BALSA data (https://balsa.wustl.edu/) platforms (Glasser et al., 2016a).

How are these new neuroanatomical data fitting into- or complementing- other alternative scientific strategies to achieve brain mapping? I will consider several ongoing extraordinarily ambitious projects dedicated to reconstructing the brains of mammals by tackling one of their essential building blocks: The neurons (Shillcock et al., 2016). Among them, the BigNeuron initiative project (http://bigneuron.org) provides neuronal reconstruction algorithms in one open-source platform, enabling researchers to compare and test their own algorithms with a large set of image slices. In turn, the database Neuromorpho.org (http://neuromorpho.org/) is the largest collection of publicly accessible 3D neuronal reconstructions with about 34,000 neurons. Finally, the Blue Brain project (http://bluebrain.epfl.ch/), aiming to build biologically detailed digital reconstructions and simulations of the rodent- and ultimately the human brain-, published a first draft reconstruction of a portion of juvenile rat somatosensory cortex from a collection of ~2,000 biological reconstructions (Markram et al., 2015). In this impressive work, authors algorithmically reconstructed the detailed neuronal anatomy and physiology by using cellular and synaptic organizing principles and then classifying the neurons in terms of well-established morphological types and reconstructing the connectivity between them. Concerning the human brain, however, the scenario is, unfortunately, worse. To date, there are two large-scale projects under development: (i) the European Human Brain project (https://www.humanbrainproject.eu/), whose main objective is to create a HBP research infrastructure by generating six cutting-edge Information ICT Platforms and (ii) the US BRAIN initiative (https://www.whitehouse.gov/BRAIN), undoubtedly one of the former US Administration's “Grand Challenges,” but that is still in a preliminary stand-by situation at the NIH.

Considering these reasons, the new data obtained by Glasser and colleagues and their future applications represent a real breakthrough in the field of human brain mapping.

## Counting synapses in living human brains

The pathogenic events of many mental and neurodegenerative diseases are triggered by reductions in the number of synapses (Selkoe, 2002; Scheff et al., 2006; Bernardinelli et al., 2014; Robinson et al., 2014). Serial reconstructions from ultrathin sections and their examination by transmission electron microscopy (TEM) have been for decades (since the 1950s) the only way to examine the number of synapses per volume or synapse distribution and size. Serial sectioning TEM has been a well-established technique to obtain 3D data of brain tissue in animal models (Stevens et al., 1980); however, it is technically demanding and time-consuming, with the added difficulty of having long series of correlative ultrathin sections and precluding the reconstruction of large volumes of tissue. After working with *Drosophila* brain serial TEM sections for years at the Cajal Institute, this author can fully certify all these constraints (Acebes and Ferrús, 2001). However, several years ago, a combination of focused ion beam milling and scanning electron microscopy (FIB/SEM) was revealed as a suitable technique for studies on experimental animals, allowing automatic serial sectioning of large tissue volumes to be later reconstructed in 3D. Indeed, FIB/SEM has resulted in new avenues for 3D synapse and spine reconstructions (Merchán-Pérez et al., 2009; Bosch et al., 2015) and has also improved the visualization of cells and tissues (Drobne, 2013).

There are only a few quantitative TEM studies in humans, notably hampered by technical issues and most of them are related to tissue sample quality and availability. Thus, whereas ultrastructural preservation of post-mortem human brain tissue is often not adequate, biopsy material is more suitable and allows for the application of quantitative EM methods (Alonso-Nanclares et al., 2008, Figure 1C). Biopsy, however, presents the limitations of having to obtain only small samples and is restricted by medical and also ethical parameters. For these reasons, indirect methods have been employed to estimate synaptic density in human tissues, relying on synaptic markers (e.g., counting synaptophysin-immunoreactive puncta) and using conventional light and/or confocal microscopy (Arendt, 2009). Interestingly, 3 years ago, a team of researchers tested the abovementioned FIB/SEM to study the ultrastructure of the synaptic organization of human brain cortical areas from AD patients (Blazquez-Llorca et al., 2013). Remarkably, despite of the promising quantitative results presented in this paper, there are no further studies employing FIB/SEM technology to count synapses in human brain tissues.

This scenario may change thanks to the recent work of Finnema and collaborators reporting the use of positron emission tomography (PET) to count synapses (Finnema et al., 2016). In the late 1970s and early 1980s, the use of PET imaging in living humans enabled *in vivo* quantification of a wide range of proteins including brain receptors, transporters and enzymes among others (for a review, see Jones et al., 2012). Here, the major achievement of Finnema and collaborators has been to combine this well-established PET methodology with the development and deep characterization of a radioligand named [^11^C]UCB-J which binds to SV2A, a ubiquitous brain isoform of SV2 located in the presynaptic vesicle membrane (Bajjalieh et al., 1994). For the first time, their achievement has allowed for the quantification of synaptic densities in a living human brain (Figure 1D).

Key details for which I consider this paper as extraordinarily compelling:

1. The authors validated the synaptic-radioligand in a PET assay on a primate brain (baboon) by comparing regional densities of SV2A vs. synaptophysin, a bona fide synaptic marker currently employed in inmmunohistochemistry (see above). They also performed *in vitro* binding assays and Western blotting to confirm the *in vivo* data.
2. This first-in-human imaging study demonstrates that [^11^C]UCB-J had excellent imaging properties in the human brain, while being minimally invasive. Besides, SV2A-PET imaging over 10 healthy humans yields similar results when compared with the baboon brain, confirming that [^11^C]UCB-J represents a quantitative marker of synaptic densities.
3. To confirm that [^11^C]UCB-J binds specifically to SV2A, they performed a pharmacological displacement study employing the SV2A-selective anticonvulsant drug Levetiracetam. The drug substantially decreased binding of [^11^C]UCB-J in SV2A-rich brain regions by 90–120 min.
4. Finnema and colleagues evaluated whether [^11^C]UCB-J is a suitable tool to monitor synaptic loss. To this end, [^11^C]UCB-J binding was used in three patients with temporal lobe epilepsy (TLE) revealing unilateral synaptic loss. This is the first proof of concept obtained in human patients showing how changes in synaptic density can be monitored non-invasively in a living brain.

All these facts place [^11^C]UCB-J SV2A PET imaging as a promising *in vivo* approach for research, clinical diagnosis and therapeutic monitoring in psychiatric and neurological disorders in which synapse number is severely compromised, being particularly relevant to early stages of Alzheimer's disease patients.

## The final goal: to join anatomy and function

A new scientific avenue is now open for human brain mapping and reconstruction. In turn, a synapse counting *in vivo* method will help to be one step ahead of pathologies at earlier disease stages. One of the future tasks will be to characterize brain functional organization by combining non-invasive imaging techniques, molecular biology, genetics, computational models of neuronal networks and the finest description of connectivity and synaptology. Undoubtedly, the challenge of knowing our own brain deserves such an immense effort.

## Author contributions

The author confirms being the sole contributor of this work and approved it for publication.

## Funding

This publication was supported by Fundación CajaCanarias, Fundación La Caixa and the Spanish National Programme for Research aimed at the Challenges of Society (DPI2015-66458-C2-2-R, MINECO) to AA.

### Conflict of interest statement

The author declares that the research was conducted in the absence of any commercial or financial relationships that could be construed as a potential conflict of interest.
